# Global medical education partnerships to expand specialty expertise: a case report on building neurology clinical and research capacity

**DOI:** 10.1186/1478-4491-12-75

**Published:** 2014-12-30

**Authors:** Mark Kaddumukasa, Elly Katabira, Robert A Salata, Marco A Costa, Edward Ddumba, Anthony Furlan, Angelina Kakooza-Mwesige, Moses R Kamya, James Kayima, Chris T Longenecker, Harriet Mayanja-Kizza, Charles Mondo, Shirley Moore, Svetlana Pundik, Nelson Sewankambo, Daniel I Simon, Kathleen A Smyth, Martha Sajatovic

**Affiliations:** Department of Medicine, School of Medicine, Makerere University College of Health Sciences, Kampala, Uganda; Department of Medicine, Case Western Reserve University School of Medicine and University Hospitals Case Medical Centre, Cleveland, OH USA; Cardiology, Case Western Reserve University School of Medicine and University Hospitals Case Medical Centre, Cleveland, OH USA; Department of Medicine, Uganda Martyrs University, Postgraduate Medical Education Nsambya, Kampala, Uganda; Department of Neurology, Case Western Reserve University School of Medicine and University Hospitals Case Medical Centre, Cleveland, OH USA; Department of Paediatrics, School of Medicine, Makerere University College of Health Sciences, Kampala, Uganda; School of Nursing, Case Western Reserve University, Cleveland, OH USA; Department of Neurology, Case Western Reserve School of Medicine, Louis Stokes VA Medical Centre, Cleveland, OH USA; Makerere University College of Health Sciences, Kampala, Uganda; Department of Epidemiology and Biostatistics, Case Western Reserve University, Cleveland, OH USA; Department of Psychiatry and Department of Neurology, Case Western Reserve University School of Medicine and University Hospitals Case Medical Centre, Cleveland, OH USA

**Keywords:** Medical education, Neurology, Neurological disorders, Research education, Global health, Uganda, sub-Saharan Africa, Cardiovascular disease

## Abstract

**Background:**

Neurological disorders are a common cause of morbidity and mortality in sub-Saharan African, but resources for their management are scarce. Collaborations between training institutions in developed and resource-limited countries can be a successful model for supporting specialty medical education and increasing clinical and research capacity.

**Case report:**

This report describes a US National Institutes of Health (NIH) funded Medical Education Partnership Initiative (MEPI) to enhance expertise in neurology, developed between Makerere University College of Health Sciences in Kampala, Uganda, and Case Western Reserve University School of Medicine in Cleveland, OH, USA.

This collaborative model is based on a successful medical education and research model that has been developed over the past two decades. The Ugandan and US teams have accumulated knowledge and 'lessons learned' that facilitate specialty expertise in neurological conditions, which are widespread and associated with substantial disability in resource-limited countries. Strengths of the model include a focus on community health care settings and a strong research component. Key elements include strong local leadership; use of remote technology, templates to standardize performance; shared exchanges; mechanisms to optimize sustainability and of dissemination activities that expand impact of the original initiative. Efficient collaborations are further enhanced by external and institutional support, and can be sequentially refined.

**Conclusion:**

Models such as the Makerere University College of Health Sciences - Case Western Reserve University partnership may help other groups initiate collaborative education programmes and establish successful partnerships that may provide the opportunity to expand to other chronic diseases. A benefit of collaboration is that learning is two-directional, and interaction with other international medical education collaborators is likely to be of benefit to the larger global health community.

**Electronic supplementary material:**

The online version of this article (doi:10.1186/1478-4491-12-75) contains supplementary material, which is available to authorized users.

## Background

Africa faces complex health issues that involve a wide range of social, political, economic, and biomedical factors that can be optimally addressed by innovative and coordinated approaches. Partnerships between training institutions in developed and developing countries can support medical education in resource limited settings (RLS) [1]. These collaborations are an effective way of harnessing multiple disciplinary perspectives to tackle complex issues in health research and training, such as attainment of the Millennium Development Goals in RLS [2]. Although initiating these partnerships can be challenging, once successfully established, they can be of significant mutual benefit. With a long-term commitment by both partners, these initiatives can become self-sustaining, and support development of much-needed related training programmes in other RLS [3, 4].

This report describes a Medical Education Partnership Initiative (MEPI) to enhance expertise in neurology, developed between Makerere University College of Health Sciences (MakCHS) in Kampala, Uganda, and Case Western Reserve University (CWRU) School of Medicine in Cleveland, OH, USA. These institutions have a successful medical partnership model that may help guide implementation of other global health initiatives. The MakCHS-CWRU model has been developed over two decades, and teams have accumulated expertise in refining an approach that is efficient and practical. Review of the incremental elements of building the partnership informs our summary recommendations and includes 'lessons learned' that are critical to a generalizable partnership model.

## Case description

### The emerging health target of chronic noncommunicable diseases

Chronic, noncommunicable diseases (NCDs) are increasing around the world, with disproportionate burden on countries with limited resources. Eighty percent of deaths from NCDs occur in developing countries, and 29% of deaths from NCDs in these countries happen among people under age 60, compared to 13% in high-income countries [5]. NCDs, such as cardiovascular disease (CVD), are expected to cause three quarters of the disease burden in low- and middle-income countries (LMICs) by 2030 [6]. Nervous system conditions such as epilepsy, dementia, and mental disorders, are also important causes of NCD-related disability [7]. Unless action is taken to lessen the toll, the World Bank estimates that NCDs will cost the global economy about $35 trillion from 2005 to 2030 [6]. Reflecting concern over the looming NCD epidemic, health representatives from 200 countries recently met and voted unanimously to focus their attention and resources on NCDs. Consistent with this global initiative, and building on the significant infrastructure that has been established for infectious diseases, the MakCHS-CWRU team worked with colleagues at Mulago Hospital and the Joint Clinical Research Centre in Uganda to expand clinical and research capacity to include CVD and neurological conditions.

### The problem of increasing disease burden and lack of human resources to address the problem

Neurological disorders cause 16.8% of deaths in LMICs, compared with 13.2% of deaths in high-income countries [8, 9]. Neurological disorders contributed to 92 million disability-adjusted life years (DALYs) in 2005, and this burden is projected to increase by approximately 12% worldwide by 2030 [8].

Epilepsy is one of the most common serious neurological disorders worldwide, affecting an estimated 50 million people. In Uganda, epilepsy is the most common neurological disorder, with an estimated prevalence of 2 to 5 persons per 1,000 people in the country. It has been reported that the number of new epilepsy cases in Uganda in 1 year was 156 among 100,000 people [10]. Epilepsy is especially prevalent in onchocerchiasis endemic areas, with rates as high as 15 to 20 cases per 1,000 people in the Kabarole and Nebbi districts in Uganda [11].

With respect to stroke, data specific to Uganda is sparse. Reported 30-day mortality among patients admitted in Mulago with stroke is unacceptably high at 43.8% (unpublished data by Levi Kwarisiima). Data on other neurological disorders, such as Parkinson’s disease, dementia, and muscular dystrophies remain to be investigated.

Despite the growing importance of neurological diseases as a common cause of morbidity and mortality, and the ongoing efforts to determine the incidence, prevalence and burden of neurological disorders, human resources and services for dealing with neurological disorders are disproportionately scarce in Uganda. The median number of neurologists per 100,000 population is about 0.03 in Uganda, compared to 2.96 in high-income countries [12]. The MakCHS has two practicing paediatricians specializing in neurology, who support training for residents and undergraduates [13], as well as nurses in neurology-related management, while the adult neurology ward relies on a team of five physicians, four of whom are yet to be specifically trained in neurology. Teams practice in the city centre, and coverage of rural areas by neurologists is virtually non-existent. Among the seven practicing neurology medical personnel in Uganda, three have had specialized general neurology fellowship training and certification for general neurology. None have had subspecialty neurology training focused on expertise domains such as stroke, epilepsy, or neurological conditions of later-life. There is an urgent need for neurology training to appropriately address this deficit.

Throughout Africa, and Uganda in particular, it is vitally important to develop capacity in prevention and management of neurologic conditions in children and adults, from primary health care to the subspecialist level [13, 14]. The shortage of specialists in neurological diseases calls for training a critical mass of leaders in sub-Saharan Africa who can then sustain independent research, teaching, and clinical careers, and contribute to the formulation of policy for the prevention and care of neurological disease.

Research priorities in Uganda are being re-aligned to address the issue of increase in NCDs. Up until recently, the majority of research in Uganda has been on infectious diseases, especially HIV, malaria, and tuberculosis. Not surprisingly, most medical specialists and researchers have tracked into infectious disease. However, the medical profession in Uganda is currently undergoing the establishment of subspecialties. An NCD department has been established in the Ugandan Ministry of Health to address these gaps and advise on resource allocation and research priorities. Taken together, these new efforts will help Uganda to develop programmes and legislation related to neurological disorders, with the purpose of building capacity in both neurology training and research.

### Putting experience into action for neurology care and research: importance of the MakCHS-CWRU based partnership

The Uganda-CWRU Research Collaboration began in 1986 after a Presidential invitation to Frederick C Robbins, MD, CWRU Professor Emeritus and Nobel Laureate, to visit Uganda and assist the Ugandan Government with the HIV/AIDS epidemic. Dr. Robbins served with Dr. Samuel Okware, then Director of the AIDS Control Programme in Uganda, as Principal Investigator (PI) of a multidisciplinary research programme, 'International Collaboration for AIDS Research' (ICAR), funded by the National Institutes of Health in 1988. The UCRC continued to prosper, supported by ongoing US federal and other research funding. The collaborative programme has allowed Uganda’s research agenda to expand, with Ugandan investigators further seeking research opportunities using the collaborative programme infrastructure.

### The Medical Education Partnership Initiative (MEPI)

MEPI is a coordinated effort led by the Office of the US Global AIDS Coordinator (OGAC) at the Department of State, and supported by the National Institutes of Health (NIH), the Fogarty International Centre, the Office of AIDS Research (OAR), and the Office of Research on Women’s Health (ORWH) in the Office of the NIH Director and the Health Resources and Services Administration’s (HRSA) Administrator. The MEPI initiative supports medical education and research in sub-Saharan Africa, and is intended to increase the quantity, quality, and retention of graduates with specific skills to address the health needs of their national populations. The MEPI model has potential to extend its benefits beyond sub-Saharan Africa to advance care for patients and communities globally.

### A successful MEPI to address cardiovascular disease

Despite the fact that CVD is a key cause of morbidity and mortality, CVD research and clinical training has been relatively neglected in Uganda. There is also a lack of good quality epidemiologic data on CVD. The few on-site experts are in urban areas. This translates into low levels of awareness in communities, poor outcomes and suboptimal primary care responses to CVD. Faculty at MakCHS-CWRU, under the direction of PI Dr. Nelson Sewankambo recently expanded research collaborations to include CVD, and obtained funding for a 5-year MEPI project from the Fogarty International Centre and the National Heart, Lung, and Blood Institute in September of 2010. This MEPI addresses the critical shortage of national leaders in CVD, and aims to build capacity for CVD research and training in Uganda. In the CVD MEPI, CWRU provides visiting faculty, ongoing research mentorship, and targeted cardiology fellowship training.

### Building on MEPI success to expand capacity in neurological disorders

**Figure 1:**
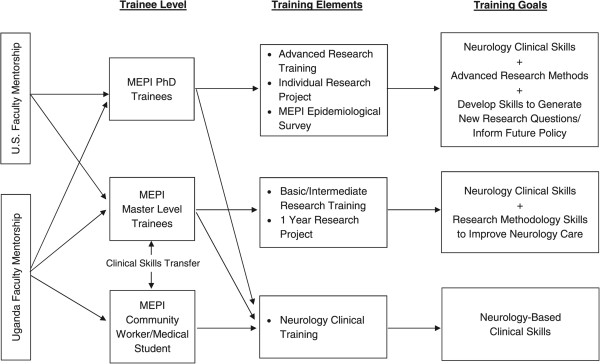
**Neurology Medical Education Parnership Initiative (MEPI) training model.**

Based upon the success of the CVD MEPI, the medical partnership team at MakCHS-CWRU, applied for and received funding for a 3-year project (30 September 2012 to 31 August 2015) from the National Institute of Neurological Disorders and Stroke (NINDS, 1R25NS080968-01), which is focused on expanding neurology research and clinical capacity. Figure 1 illustrates the partnership participants and programme goals of the Neurology MEPI programme directed by PI Dr. Elly Katabira at MakCHS. Building on the CVD MEPI model, the MakCHS-CWRU team has developed an educational programme for neurology research and training among medical students, residents and faculty.The established MakCHS-CWRU relationship provided a strong foundation for training, support and collaboration between the University Hospitals Case Medical Center Neurological Institute and MakCHS, neurology unit. The Neurology MEPI tapped into the expertise of a network of clinicians and health scientists at CWRU, with specialized foci that include an epilepsy assessment and monitoring unit, certified stroke centre, and programme for post-stroke neurology rehabilitation. The CWRU mentoring largely focused on support of PhD and Master’s level candidates who were trained in neurological clinical skills and research methods (Figure 1). The MakCHS faculty efforts targeted PhD, Master’s level candidates, and medical students and health workers in the community (Figure 1). The orientation of the pedagogical community was interactive, practically-focused and hands-on with iterative contact. While a main goal of the Neurology MEPI was to promote research and clinical training in neurology, the MEPI also included a cross-sectional epidemiological survey to assess the prevalence of neurological diseases and disease risk factors in Uganda. The overall product is intended to boost the clinical and research workforce in neurology, and provide the much-needed epidemiological data that will inform future research and clinical planning.

### Neurology MEPI working model details

Training takes place in the enhanced infrastructure at Makerere Mulago and Nsambya teaching hospitals, in the community (where PhD students enroll cohorts for their studies), and at the collaborating institutions in a mentored environment. Faculty at MakCHS and Uganda Martyrs Hospital serve on a programme implementation committee (PIC), which coordinates communication among MEPI trainees and faculty, day-to-day running of the programme, selection of trainees and implementation of research surveys. The trainee selection process is competitive, and involves solicitation for candidates, an application process using a standardized scoring rubric (see Additional file 1: Figure S1), and PIC/faculty consensus on candidate selection. Master’s and PhD trainees are selected among postgraduate students who have completed medical school. The Neurology MEPI has enrolled two PhD level trainees, and ten residents in the Master’s Degree training programme (including trainees from programmes in internal medicine, paediatrics and psychiatry). All trainees have satisfactorily met their targeted goals of project implementation and are in various stages of reporting their project strategy or results. Table 1 illustrates trainee research projects that feature newly acquired clinical skills and neurology-focused data collection. No trainees have terminated participation in the programme.The MakCHS and Nsambya hospitals provide the neurology teaching staff who act as local mentors to students, other trainees and junior faculty. Efforts are geared at developing a qualified physician or paediatrician with a keen interest in neurology and well equipped to develop inquiry, analysis and action into problems of neurological disorders (Figure 1). Each trainee is allocated two local mentors who participate in training and research-related activities.

**Table 1:** **Neurology MEPI Trainee Projects**

Project title	Degree sought	Year started
Factors Associated With Stroke Among Hypertensive Patients: A Case Control Study Among Patients Admitted In Urban Hospitals In Kampala, Uganda.	MS	2012
Depression In Post-Stroke Patients Attending A Peri-Urban Hospital In Kampala Uganda.	MS	2012
The Prevalence And Factors Associated With Multiple Anti-Epileptic Drug Requirement In Children.	MS	2012
The Association Between Immune Status And HIV Depression With Pain Comorbidity.	MS	2012
Prevalence And Risk Factors Of ADHD Among Children Attending Neurology And Psychiatric Clinics At Mulago National Referral Hospital.	MS	2013
Prevalence Grading And Associated Risk Factors For Peripheral Neuropathy Among Diabetic Patients Attending Mulago Diabetic Outpatient Clinic.	MS	2013
Prevalence And Factors Associated With Lower Serum Vitamin B12 Among Diabetic Patients Attending The Diabetic Clinic - Mulago Hospital.	MS	2013
Sodium Intake In Post-Stroke Patients, And Its Influence On BP Control And Stroke Outcomes In Uganda.	PhD	2012
Epidemiology, Risk Factors, Prevention And Outcome Of Stroke In Children With Sickle Cell Anemia.	PhD	2012

The University Hospitals Case Medical Center Neurological Institute provided two additional mentors, who were selected based on their specialty interest and expertise. This exposes trainees to a diverse experience of supervision and teaching. Clinical foci include acute stroke care, epilepsy management training, electroencephalogram (EEG) interpretation skills, and peripheral nerve and neuromuscular studies.

The Neurology MEPI programme emphasizes faculty-trainee interaction. This mutually beneficial relationship helps to ensure capacity-building at the local institutions and to facilitate sustainability. Given the geographic distance between regional and international sites, teams rely heavily on web-based technology for information exchange. Web-based routine communication is supplemented with in-person faculty and trainee site exchange visits and education. Academic exchange in the MakCHS-CWRU partnership is two-directional, with approximately two trainees and/or faculty cross-visiting on a yearly basis.

One of the innovations of this collaboration has been shared videoconferencing for grand rounds between MakCHS and the CWRU Neurology Institute. Two grand rounds included students, residents and fellows from both the Ugandan and US sites. Trainees had the opportunity of learning from the experts in neurology, and being able to interact and ask questions of experts and their international colleagues. Grand rounds have been shared with other local Medical Education for Equitable Services to all Ugandans (MESAU) Consortium participating universities joined thorough Internet linkage to the videoconferencing.

### Neurology training at the community level

Like the CVD MEPI, the Neurology MEPI addresses the issue of limited expertise in rural settings by using the novel Community-Based Education, Research and Service (COBERS), which trains medical students and health workers in awareness, assessment and basic management of neurological disorders. In contrast to a traditional model of hospital-based lectures, where most of the contact is with patients receiving tertiary care for complications, COBERS is a model of community care at the local level. Students are trained to be responsive to stroke prevention and neurological disorders in primary care. The selected training sites are lower level (district) peripheral health centres that serve a population of approximately 100,000 and MakCHS medical students use these sites for their community placement training. COBERS students are trained in stroke risk factor screening, community education, neurological clinical examinations, diagnostic tests and subsequent patient care. The community-based peripheral centres are an efficient and practical approach for general health worker training in neurology care. Thus far in the Neurology MEPI, 97 health worker trainees (medical doctors, nurses, clinical officers) from various COBERS community rural health units have received specific neurology-related training with emphasis on stroke and its prevention within the community. Topics included stroke prevention, early detection and appropriate referrals for patients from these communities, as well as an overall aim of increasing neurology competence.

MEPI community clinical mentors supervise students during their rotation, and offer administrative and medical services within these community health centres. The health workers at these community level health centres are qualified medical doctors and nurses who provide general medical care for their communities and provide problem-based approach teaching for the students. Clinical supervisors reinforce clinical skills and medical history taking with emphasis on neurology during the community rotations. The community mentors are supported by MakCHS faculty tutors who visit and participate in student training and supervision.

Like training with PhD and Master’s level candidates, the pedagogical community training is designed to be interactive and iterative. This early introduction and training for undergraduate medical students is intended to demystify neurology as a subject usually considered difficult by undergraduates. The training curriculum introduces the principles underpinning the anatomical, physiological and clinical basis of neurological disorders. The training imparts clinical skills relevant to the diagnosis of neurological disorders and their subsequent management.

The main course objectives include:

-  Understanding the anatomical and physiologic basis of neurological disorders
-  Understanding basic concepts of neurological disorders, for example: neuropathic pain, seizures, movement disorders, stroke, voluntary muscle and neuromuscular disorders, dementia, and so on.
-  Reinforce the art of history taking in neurological disorders
-  Initiate and consolidate the mastery of eliciting physical signs in the diagnosis of neurological disorders
-  Use pattern recognition and analytical methods in making neurological diagnosis, based on information collected from the clinical process
-  Describe trends in neurologic disease occurrence and propose approaches to prevention, based on epidemiological data from the health information system.

Taken together, the community curriculum provides a strong basis for undergraduates to appreciate neurology and use skills to prevent, assess and manage neurological conditions at the primary care level.

### Conducting epidemiological research to inform future practice and investigation

The Neurology MEPI epidemiological survey is based in a COBERS network and offers graduate students and faculty an opportunity to collect neurology-focused epidemiological data and apply their new clinical and research skills. The recently completed survey identifies prevalence, risk factors and knowledge/attitudes with respect to neurological disorders, and is intended to provide data for planning of neurology public health programmes. The cross- sectional survey was conducted in two sub-counties of Mukono Town Council and Ntenjeru subcounty over a 6-month period. These subcounties were classified as rural or urban, based on information from the district administration and the current level of urbanization. Approximately 3,000 adults were recruited from the list of households randomly generated from a census database at each site. The modified WHO survey questionnaire was used as a screening tool, and those found to suggest occurrence of neurological disorder were seen by two neurology physicians to confirm the presence of the disorder. The survey complemented PhD student work investigating stroke and dietary salt among participants in the community and prevalence of stroke among sickle cell sufferers. This has also offered an opportunity for trainees to develop good clinical practice (GCP) and human research ethics training while conducting community research. While survey findings have not yet been analyzed, it is expected that they may help guide National Ministry of Health formulation of strategies for prevention of neurological conditions, which now heavily impact the citizens of Uganda. For example, peripheral neuropathy, a late-stage progression of metabolic disease, is a first clinical presentation among many Ugandan patients with diabetes. Neurology MEPI survey findings may facilitate increased attention to risk factors at a point where interventions may still be effective. Other examples of prevention include control of hypertension related to stroke prevention, appropriate use of anticonvulsants in epilepsy, and recognition and treatment of psychiatric disorders.

## Discussion

As many neurological conditions result in extended years of disability, often beginning at a very young age, allocation of resources for neurological care and treatment in sub-Saharan Africa must be a public health priority. As Berkowitz [15] recently noted, there are 3 neurologists per 10,000,000 of the population in low-income countries, some of which do not have even a single neurologist. Training the next generation of scientists and clinicians to study and treat patients with neurological disorders thus invests in the future of national health care that can help LMICs manage increasingly common NCDs.

Craven [16] recently summarized progress in neurology training in Africa that included the first regional teaching course for neurology trainees in 2008, a collaboration between the World Federation of Neurology (WFN), the European Federation of Neurological Societies and the International Brain Research Organization (IBRO) held in Dakar, Senegal. WFN hopes to increase the number of neurologists working in Africa by 10 each year.

In 2008, the first neurology residents in Ethiopia completed their training at Addis Ababa University [16] and in 2009 Ethiopa had 14 neurologists (for a population of 80 million). Educational efforts that included collaboration between US neurologists and the Addis Ababa University Faculty of Medicine Black Lion Hospital in Ethiopia have resulted in a self-sustaining neurology training programme [17].

Training students to become leaders is a long-term objective beset by many challenges. We acknowledge that the Uganda Neurology MEPI programme is still in the implementation stage and we do not have longitudinal data to demonstrate that the programme has produced independent scientists and clinicians who regularly manage neurological disease. However, interim progress thus far seems successful, and the MakCHS-CWRU Neurology MEPI has provided multiple opportunities for lessons learned, from which we draw several recommendations:

1. Leadership is key to initiating and sustaining change. Leadership and guidance of the Neurology MEPI collaboration come from multiple sources. Ugandan and US faculty were selected based upon trainee needs, anticipated challenges and expertise at each of the collaborating sites. A successful partnership requires strong leadership to make decisions, take appropriate risks, and solve problems. During programme development, the MakCHS-CWRU team formulated a common mission, and agreed upon expectations and goals for each partner.
2. Teamwork includes shared responsibility, mutual respect, open communication, compromise and capitalizing on partner strengths for faculty-mentored training. Our partnership leveraged institutional and individual strengths. CWRU provided neurology expertise, seasoned multidisciplinary mentorship, and research methodology skills. Each trainee has multiple mentors at each site (with a designated primary mentor at each site) in order to take advantage of complementary mentor strengths. Makerere University College of Health Sciences and Nsambya School of Postgraduate Medical Education provided the required environment, faculty and residents with interest in neurology training.
3. *Use of remote technology* establishes good communication and facilitates real-time decision-making. The Mak**C** HS-CWRU teams hold regular conference calls with web-facilitated participation using low or no-cost conferencing software. E-mail correspondence between leadership and mentees/mentors facilitates sequential refinement of protocols and optimizes responses to Institutional Review Board (IRB) recommendations. Careful planning ahead for site visits helps make in-person visits efficient and effective.
4. *Templates and protocols* establish standards for programme implementation, and the MakCHS-CWRU team makes extensive use of re-usable formats. Examples of such templates in the Neurology MEPI include 1) a standardized scoring rubric for trainee project applications, 2) standardized teleconference agendas and 3) standardized training modules for community education. Additional file 1: Figure S1 illustrates a scoring rubric for trainee applications. Proposals are reviewed by multiple faculty and scored individually. The PIC considers both mean and individual scores in the candidate selection process. Internet-based teleconferences follow a standardized agenda that tracks trainee progress, facilitates input from site mentors, and provides a built-in platform for managing regulatory and reporting requirements. The training module for undergraduates during their pre-clinical community training includes development of standardized cases, questions, and content on neurology- related conditions which are similar at all community sites. The COBERS curriculum ensures that minimum standards are maintained, and ideally will encourage interest in neurology in young students.
5. *Shared exchanges*. Trainee and faculty site visits help meet overall programmatic goals. Visiting experts provide motivation, confidence-building and guidance for students and junior faculty. Trainees visiting sites outside of their typical settings can identify new areas of special focus/interest and customize training experiences. Trainees can then serve as models to other more junior colleagues, and pass along new knowledge and skills. Staff and faculty involved in the CVD MEPI were extremely important in helping the Neurology MEPI team establish successful procedures quickly and reduce duplicative effort. It is critical that programmes explicitly emphasize team-learning, trainee interaction, and the expectation that specialty clinical and research expertise are valuable and readily-acquired skills that can broadly improve care for patients and their community.
6. *Sustainability*. Supporting skilled PhD and Master’s level graduates to develop their own programmes and train additional support staff (such as community workers and new students) will extend their reach in patient care settings. The Master's-level programme included participation in the training of undergraduate students in developing clinical skills such as performing a neurological exam. The COBERS model can thus expand the impact of MEPI geographically and at an earlier point in the care continuum. Health care workers who see preventative care in action are more likely to adopt these practices in their own clinical practice, and the ripple effects of improved standards of care are likely to lead to better health outcomes. The integration of the neurology training module into the COBERS curriculum with student orientation will serve to maintain sustainability of this training, and faculty of MakCHS and Nsambya hospitals will continue to offer support and mentoring in neurology- related activities. Manuscripts and reports generated thus far have provided information on neurological disease prevalence and burden that has not been previously described. Finally, both PhD students are now junior faculty within the College of Health Sciences, Departments of Paediatrics and Internal Medicine, and participate in support and mentoring of trainees and colleagues. This will ensure neurology research and training sustainability when the MEPI programme eventually ends.
7. *Dissemination*. Making research findings to the relevant health planning authorities and government is important in ensuring sustainability and continuous improvement in neurology on a larger scale. We are highly invested in expanding the programme beyond urban areas to make the overall success meaningful, and plan to present the programme to the Ugandan Ministry of Health and at international meetings. High academic output, rigorous performance evaluation and continuous improvement are critical. In the end, positive endorsement by successful trainees will speak to the success of MEPI.

### Limitations

The Neurology MEPI programme is still in the implementation stage and our discussion/conclusion is based upon this interim data. There is also very limited data that exists to inform clinical care of individuals with neurological conditions in Uganda, and the similarly scarce human resources to address these problems.

## Conclusion

This Neurology MEPI programme is expected to expand capacity for neurology care and research in Uganda, and eventually lead to improved health outcomes for patients and families. Other expected advances include establishment of neurology specialized centres in Ugandan hospitals and training institutes, and new and additional research in NCDs. Neurology community training provides previously unavailable education to medical students in areas without specialty neurologists. Additional benefits include better patient care services by trained and qualified staff, and development of preventive policies and screening protocols based on new epidemiological studies in neurological diseases.

As demonstrated by the long-standing and multi-year MakCHS-CWRU partnership, external and institutional support is essential to developing efficient collaborations that can be refined sequentially. Templates, protocols and remote method training can help successfully overcome geographical and other challenges.

In the future, we hope that MEPI teams, such as the MakCHS-CWRU partnership, can support other groups planning to initiate collaborative partnerships. Furthermore, interaction with other international medical education collaborators is likely to be of substantial benefit to the larger scientific and health care community, and new funding opportunities by NIH, Fogarty or other institutions for education and research can facilitate further advances in global health.

## Electronic supplementary material

Additional file 1: Figure S1: Neurology Medical Education Partnership Initiative (MEPI) scholarship programme scoring template. (PDF 214 KB)

## Abbreviations

COBERS: Community-Based Education, Research and Service
CVD: cardiovascular disease
CWRU: Case Western Reserve University, Cleveland, OH, USA
DALY: disability-adjusted life years
EEG: electroencephalogram
GCP: good clinical practice
HRSA: Health Resources and Services Administration
IBRO: International Brain Research Organization
ICAR: International Collaboration for AIDS Research
IRB: Institutional Review Board
LMIC: low- and middle-income countries
MakCHS: Makerere University College of Health Sciences, Kampala, Uganda
MEPI: Medical Education Partnership Initiative
MESAU: Medical Education for Equitable Services to all Ugandans
NINDS: National Institute of Neurological Disorders and Stroke
NCDs: noncommunicable diseases
NIH: National Institutes of Health
OAR: Office of AIDS Research
OGAC: Office of the US global AIDS coordinator
ORWH: Office of Research on Women’s Health
PI: Principal Investigator
PIC: programme implementation committee
RLS: resource limited settings
UCRC: Uganda-CWRU Research Collaboration
WFN: World Federation of Neurology.
