# Supplementary material for: Global medical education partnerships to expand specialty expertise: a case report on building neurology clinical and research capacity
Source: Hum Resour Health. 2014 Dec 30;12:75. doi: 10.1186/1478-4491-12-75 (PMC4531526; doi:10.1186/1478-4491-12-75)
Supplement: Supplementary file 1 — Additional file 1: Figure S1: Neurology Medical Education Partnership Initiative (MEPI) scholarship programme scoring template. (PDF 214 KB) [file 12960_2014_480_MOESM1_ESM.pdf]

Applicant Name:

Reviewer:

--- PLEASE DOUBLE-CLICK SCORING DOMAINS TO ENTER YOUR SCORES ON AN EXCEL FILE---

1. Significance of the project (25 pts)
2. Relevance to theme of grant (25 pts)
3. Innovation (10 pts)
4. Overall methods (25 pts)
5. Measures and assessments (10 pts)
6. Ethics and human subjects (5 pts)

SCORE | **0.00** |

COMMENTS:

-- Reviewers please do not score funding recommendation--

RECOMMENDATION: _____ **fund** ____ don't fund

MEPI Proposal Total Score (Categories 1-6) Benchmarks:

100	highest possible score, no flaws
75	strong proposal, needs some modifications
50	minimally acceptable proposal, needs major modifications
25	weak proposal with multiple flaws
0	worst possible score, not fundable
